# The effect of complex intramural microstructure caused by structural remodeling on the stability of atrial fibrillation: Insights from a three-dimensional multi-layer modeling study

**DOI:** 10.1371/journal.pone.0208029

**Published:** 2018-11-28

**Authors:** Riqing Chen, Cheng Wen, Rao Fu, Jianning Li, Jian Wu

**Affiliations:** Institute of Biomedical Engineering, Graduate School at Shenzhen, Tsinghua University, Shenzhen, China; Universiteit Gent, BELGIUM

## Abstract

**Background:**

Recent researches have suggested that the complex three-dimensional structures caused by structural remodeling play a key role in atrial fibrillation (AF) substrates. Here we aimed to investigate this hypothesis using a multi-layer model representing intramural microstructural features.

**Methods:**

The proposed multi-layer model was composed of the endocardium, connection wall, and epicardium. In the connection wall, intramural fibrosis was simulated using fibrotic patches randomly scattered in the myocardial tissue of fibrotic layers, while endo-epicardial dissociation was simulated using myocardial patches randomly scattered in the fibrotic tissue of isolation layers. Multiple simulation groups were generated to quantitatively analyze the effects of endo-epicardial dissociation and intramural fibrosis on AF stability, including a stochastic group, interrelated groups, fibrosis-degree-controlled groups, and dissociation-degree-controlled groups.

**Results:**

1. Stable intramural re-entries were observed to move along complete re-entrant circuits inside the transmural wall in four of 65 simulations in the stochastic group. 2. About 21 of 23 stable simulations in the stochastic group were distributed in the areas with high endo-epicardial dissociation and intramural fibrosis. 3. The difference between fibrosis-degree-controlled groups and dissociation-degree-controlled groups suggested that some distributions of connection areas may affect AF episodes despite low intramural fibrosis and endo-epicardial dissociation. 4. The overview of tracking phase singularities revealed that endo-epicardial dissociation played a visible role in AF substrates.

**Conclusion:**

The complex intramural microstructure is positively correlated with critical components of AF maintenance mechanisms. The occurrence of intramural re-entry further indicates the complexity of AF wave-dynamics.

## Introduction

Atrial fibrillation (AF) is a progressive supraventricular arrhythmia characterized by rapid and disorderly atrial electrical activity [1]. For most patients, AF gradually evolves from acute AF to paroxysmal AF with more frequent and longer episodes, eventually reducing to persistent AF with an increase in AF stability over its progression [2]. The occurrence of AF requires triggering factors and maintenance substrates, but research addressing the mechanisms of AF remains controversial due to the variety of complex influencing factors and limitations of existing cardiac electrophysiological mapping techniques [3]. So far, no hypotheses have been able to completely explain the occurrence and maintenance of the electrophysiological mechanism for AF in human hearts [4].

Experimental and clinical studies have suggested that atrial fibrosis might be a mark of structural remodeling in AF [5–7]. Patchy fibrosis, severe interstitial fibrosis, and diffuse fibrosis contribute to cardiac arrhythmogenesis [8, 9]. The presence of transmural conduction was first demonstrated by synchronizing endocardial and epicardial records in 1993 [10]. Eckstein et al. [11] analyzed the high-resolution simultaneous endo-epicardial *in vivo* mapping data by quantifying the percentage of fibrillation waves, reporting that the breakthrough incidence and endo-epicardial dissociation degree increased with the complexity of the AF substrate. Gutbrod et al. [12] acquired optical and electrical signals of endocardium and epicardium synchronously in sheep with acute AF and correlated across imaging planes to gauge the synchrony of the activation patterns compared with paced rhythms, suggesting that AF substrates are dynamic three-dimensional (3D) structures with a range of discordance between endocardium and epicardium. A recent study by Hansen and colleagues [13] found that the intramural re-entry projected differently on endocardium and epicardium using high-resolution optical mapping in *ex vivo* preparations from diseased hearts, revealing the critical importance of better understanding the role of complex atrial microstructure in AF maintenance.

Increasing evidence suggests that the AF substrate should be more fully described as a 3D complex structure [14, 15]. In this study, we aim to propose a computational model incorporating feasible microstructural features to provide mechanistic insight into the feasibility of atrial intramural microstructure as a potential substrate for sustained AF. At present, computational models incorporating the atrial intramural microstructure are becoming more accessible. One of these novel models is a dual-layer model developed by Gharaviri and his colleagues [16], in which electrical connections between the layers could be introduced or removed at any time. Their results suggested that endo-epicardial dyssynchrony increases the stability of AF compared with the single-layer model. Unfortunately, their dual-layer model cannot reflect the true complexity of the atrial intramural microstructure. Alonso et al. [17] used a 3D slab simulation composed of up to 20 2D layers with an embedded region of microfibrosis to investigate the effect of the tissue thickness and to compare the dynamics of electrical excitation between the 2D layer and 3D slab. Although they defined the fibrosis degree by the calculation of the percolation threshold, they had no description of intramural microstructure on the vertical zonation. Zhao et al. [18] provided the first systematic computational analysis of an intact and entire 3D human atrial structure, including wall thickness, histologically-validated transmural fibrosis, and 3D myofiber orientation. Their novel 3D computational high-resolution framework can be used to define the fingerprints of AF drivers and develop new patient-specific treatments. Considering the requirement of tremendous computational resources, their atrial cellular activation model is based on the Fenton-Karma model, not biophysics-based cellular models.

According to feasible intramural microstructures derived from the published histological sections of structural remodeling in AF [19–21], we developed a multi-layer computational model. In this model, the atrial intramural microstructure was functionally split into intramural fibrosis and endo-epicardial dissociation by designing different distributions of patchy fibrosis in multi-layers for quantitative analysis.

## Methods

### Multi-layer computational model

**Fig 1:**
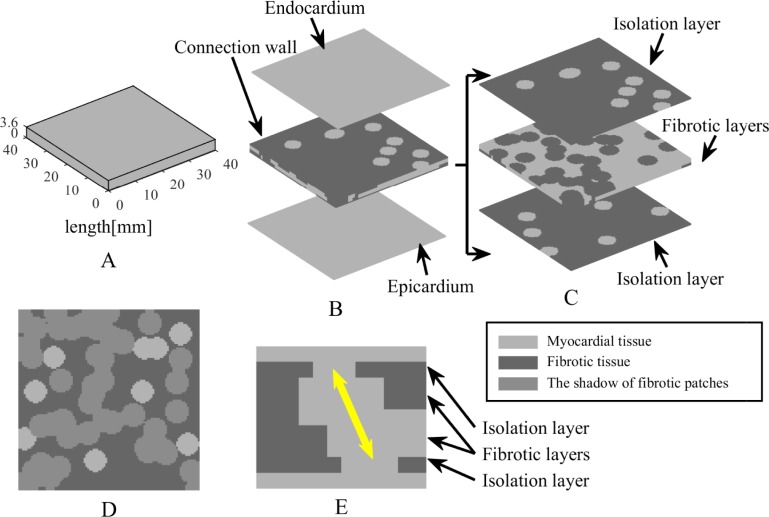
Schematic diagram of the multi-layer computational model. (A) Atrial tissue is described by a 3D slab of 40 × 40 × 3.6 mm^3^. (B) Model structure is comprised of the endocardium, connection wall, and epicardium. (C) Connection wall is subdivided into two different fibrosis distributions including isolation layers and fibrotic layers. (D) Fibrotic patches in a fibrotic layer are projected on the adjacent isolation layer. (E) The longitudinal section of model structure shows one possible path of transmural conduction (yellow arrow).

Here a multi-layer computational model was proposed incorporating some novel structure features as the proof-of-principle simplification of the anatomical complexities in the human atria. A 3D slab of 40 × 40 × 3.6 mm^3^ (Fig 1A) was used in a simplified tissue simulation, which was comprised of the endocardium, connection wall, and epicardium (Fig 1B). To be specific, the endocardium and epicardium were thin sheets of 40 × 40 × 0.4 mm^3^ without the presence of fibrosis, whereas the connection wall was designed to include two different fibrosis distributions approximately describing the complex atrial intramural microstructure in Fig 1C. The layers next to the endocardium and epicardium were thin sheets of 40 × 40 × 0.4 mm^3^ with some myocardial patches randomly scattered in fibrotic tissue forming the structure of the endo-epicardial dissociation, defined as isolation layers. Conversely, the layers next to isolation layers were thick sheets of 40 × 40 × 0.8 mm^3^ with some fibrotic patches randomly scattered in myocardial tissue forming the complexity of the intramural fibrosis, defined as thick fibrotic layers. Finally, the layer between two thick fibrotic layers was a thin sheet of 40 × 40 × 0.4 mm^3^ with fibrotic tissue automatically generated by overlaps between the fibrotic patches in thick fibrotic layers, defined as a thin fibrotic layer. Therefore, the multi-layer model was comprised of seven transmural layers including the endocardium, an isolation layer, a thick fibrotic layer, a thin fibrotic layer, a thick fibrotic layer, an isolation layer, and the epicardium from top to bottom.

All of the patches with a variety of shapes and sizes were composed of some overlapping cylindrical blobs with a certain radius. To ensure the possibility of transmural conduction, any myocardial patch in the isolation layers was not obstructed by fibrotic patches in the adjacent fibrotic layers (Fig 1D and 1E). The radius of cylindrical blobs in the isolation layers was set to 2.5 mm to guarantee that a single myocardial blob could have sufficient connectivity between the layers. Furthermore, the radius of cylindrical blobs in the fibrotic layers was set to 2.6 mm to form large patchy conduction blocks.

### Electrophysiology

The spatial spread of electric current in the atrial tissue was governed by the mathematics of the mono-domain reaction-diffusion partial differential equation with a zero flux boundary condition [22]. The transmembrane flow of the atrial myocyte was represented by the human atrial cell model of Nygren et al. [23], and the fibrotic electrophysiology was referred to Active 1 of the atrial myocyte-fibroblast coupling model of Maleckar et al [24]. The change in the number of cardiac fibroblasts could alter the electrophysiological behavior of coupled myocytes. Mathematically, the mono-domain model, combined fibrotic remodelling, is governed by the following equation:

$$\beta \left(C_{m,myo}\frac{\partial V_{myo}}{\partial t}+I_{myo}\left(V_{myo},t\right)+I_{stim}+\sum_{i=1:numf}G_{gap}\left(V_{myo}-V_{Fb}\right)\right)=\nabla \cdot \left(\sigma \nabla V_{myo}\right)$$ (1)

where *β* is the membrane surface-to-volume ratio, *C*_*m*,*myo*_ is the membrane capacitance of the myocyte, *V*_*myo*_ is the membrane potential of the myocyte, *I*_*myo*_ is the net membrane current of the myocyte, *I*_*stim*_ is the externally applied stimulus current, *G*_*gap*_ is the gap-junctional conductance between the myocyte and each fibroblast, *σ* is the conductivity tensor, and *numf* is the coupling fibroblasts number. *V*_*Fb*_ is the membrane potential of the fibroblast, governed by the following equation:

$$\frac{\partial V_{Fb}}{\partial t}=-\left(1/C_{m,Fb}\right)\left[I_{Fb}\left(V_{Fb},t\right)+G_{gap}\left(V_{Fb}-V_{myo}\right)\right]$$ (2)

where *C*_*m*,*Fb*_ is the membrane capacitance of the fibroblast, and *I*_*Fb*_ is the net membrane current of the fibroblast.

Fibrotic tissue of fibrotic layers was significantly slowly-conducting in biophysics, and isolation layers with zero conductivity of fibrotic tissue were essential for the formation of endo-epicardial dissociation. Therefore, *numf* = 3 and *σ*_*x*_ = *σ*_*y*_ = *σ*_*z*_ = 0.5*mS*/*cm* for myocardial tissue; *numf* = 45 and *σ*_*x*_ = *σ*_*y*_ = *σ*_*z*_ = 0.1*mS*/*cm* for fibrotic tissue in the fibrotic layers; *numf* = 45 and *σ*_*x*_ = *σ*_*y*_ = *σ*_*z*_ = 0 for fibrotic tissue in the isolation layers; *C*_*m*,*myo*_ = 0.05*nF*.

### Numerical calculation

The finite element method [25] was applied for simultaneous solution of the partial differential equation with ionic model equations, using hexahedral elements of 0.4 × 0.4 × 0.4 mm^3^. Thus, the complete mesh of the 3D slab was composed of 90,000 elements. To guarantee numerical stability and accuracy, all state variables of the atrial cell were updated using the forward Euler method with a fixed time step of 10 μs. The conjugate gradient method with a convergence tolerance of 10^−8^ was applied to iteratively solve the system of equations. Testing was implemented by our codes in MATLAB language running on a 3.40-GHz Core i7 workstation with 32 GB memory.

### Simulation protocol

For each simulation, intramural fibrosis degree and endo-epicardial dissociation degree were used as control variables. Intramural fibrosis degree was calculated by the following equation:

$$IFD=\frac{EleNum_{fib\_FL}}{EleNum_{tot\_FL}}\times 100\%$$ (3)

where *IFD* is the intramural fibrosis degree, *EleNum*_*fib*_*FL*_ is the element number of fibrotic area in fibrotic layers, and *EleNum*_*tot*_*FL*_ is the total element number in fibrotic layers. Similarly, endo-epicardial dissociation degree was parametrically calculated by the following equation:

$$EDD=\frac{EleNum_{fib\_IL}}{EleNum_{tot\_IL}}\times 100\%$$ (4)

where *EDD* is the endo-epicardial dissociation degree, *EleNum*_*fib*_*IL*_ is the element number of fibrotic area in isolation layers, and *EleNum*_*tot*_*IL*_ is the total element number in isolation layers. To conveniently design and control intramural fibrosis degree and endo-epicardial dissociation degree, two isolation layers had the same number of myocardial blobs and two thick fibrotic layers had the same number of fibrotic blobs.

**Fig 2:**
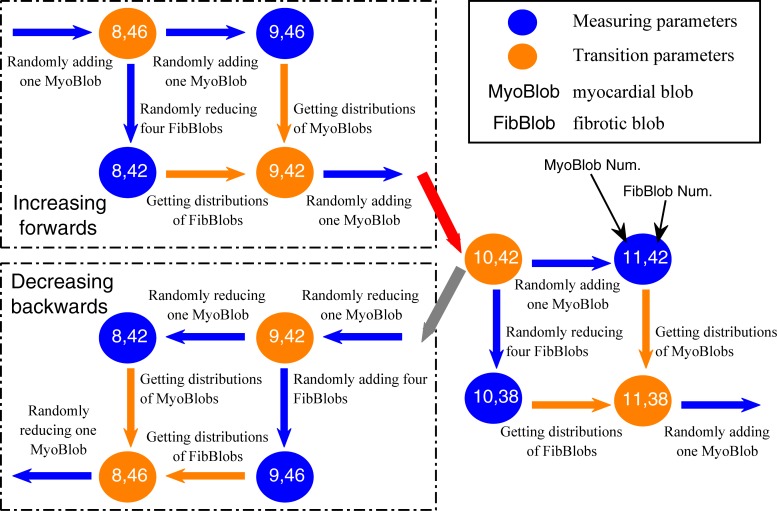
Flow chart of the proposed interrelated method on an isolation layer and its adjacent fibrotic layer. Blue arrows denote the changes of fibrotic blobs or myocardial blobs, and orange arrows denote the same distributions of fibrotic blobs or myocardial blobs. The red arrow denotes the use of the forward box, and the gray arrow denotes the use of the backward box. The measuring parameters are used to produce a series of interrelated simulations.

To quantitatively analyze the consequences of intramural fibrosis and endo-epicardial dissociation for maintaining the propagation of chaotic electrical signals during AF episodes, the following different simulation trial and control groups were applied for contrast research:

1. The stochastic group: each simulation had the randomized distributions of myocardial blobs in isolation layers and fibrotic blobs in the fibrotic layers. The number of myocardial blobs in each layer ranged from 5 to 19, and the number of fibrotic blobs in each layer ranged from 26 to 50.
2. The interrelated group generated by an interrelated method: taking some simulation with a certain number of myocardial blobs in isolation layers and fibrotic blobs in the fibrotic layers, some new myocardial blobs were added and some original fibrotic blobs were removed, or some original myocardial blobs were removed and some new fibrotic blobs were added, to produce a series of new simulations (Fig 2). Part of fibrotic blobs and myocardial blobs were the same for all simulations in an interrelated group. The simulation parameters for 13 simulations in an interrelated group were (5, 55), (6, 46), (7, 50), (8, 42), (9, 46), (10, 38), (11, 42), (12, 34), (13, 38), (14, 30), (15, 34), (16, 26), and (17, 30) (for the values within parentheses, the former denoted the number of myocardial blobs in each layer and the latter denoted the number of fibrotic blobs in each layer).
3. The fibrosis-degree-controlled group: twelve simulations had the randomized distributions but the same number of myocardial blobs in the isolation layers and the randomized distributions but the same number of fibrotic blobs in the fibrotic layers. The simulation parameters for five groups were (5, 55), (7, 38), (9, 42), (11, 46), and (13, 30).
4. The dissociation-degree-controlled group: with the same distributions of myocardial blobs in the isolation layers, twelve simulations had the randomized distributions but the same number of fibrotic blobs in the fibrotic layers. The simulation parameters for five groups were (7, 50), (9, 34), (10, 38), (11, 44), and (13, 30).

**Fig 3:**
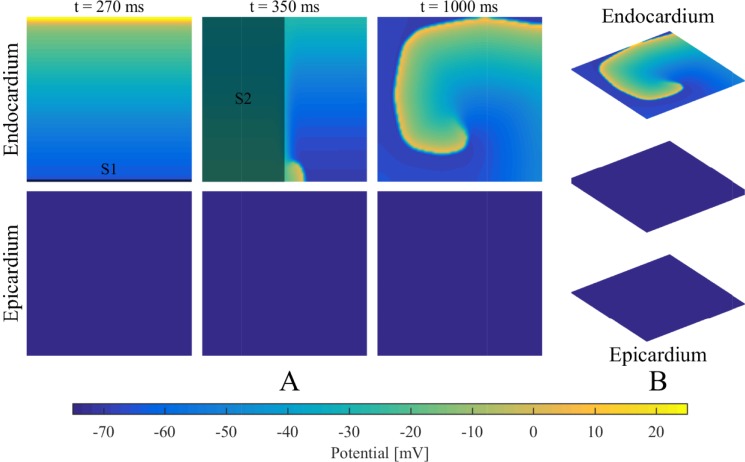
Initial condition using an S1-S2 protocol. (A) The S1–S2 protocol is used to initiate a spiral wave on the endocardium: line stimulus S1 (t = 1 ms) followed by area stimulus S2 (t = 280 ms). (2) The initial condition for the simulations.

It should be noted that intramural fibrosis degree was controlled within a certain range in terms of the relevant quantitative blobs (Table 1). To ensure the complex network had access to the atrial intramural microstructure, the distributions of myocardial blobs in the isolation layers and fibrotic blobs in the fibrotic layers needed to be adjusted to avoid gathering in the middle or scattering on the edge. As shown in Fig 3, all of the simulations adopted the same initial condition, where a spiral wave was initiated using an S1–S2 protocol only on the endocardium, as mentioned in the dual-layer model [16].

**Table 1:** The range of the intramural fibrosis degree controlled by the number of fibrotic blobs in the fibrotic layers.

Groups	Blob Num. of each fibrotic layer	Intramural fibrosis degree (%)
Stochastic group	26	21.5~22.5
	30	24.5~25.5
	34	28.0~29.0
	38	31.0~32.0
	42	34.4~35.4
	46	37.8~38.8
	50	40.7~41.7
Interrelated group	26	22.0~24.0
	30	25.0~27.0
	34	28.0~30.0
	38	32.0~34.0
	42	35.0~37.0
	46	37.5~39.5
	50	40.5~42.5
	55	44.5~46.5
Fibrosis-degree-controlled group	30	25.5~26.5
	38	32.5~33.5
	42	35.5~36.5
	46	38.0~39.0
	55	44.5~45.5
Dissociation-degree-controlled group	30	24.5~25.5
	34	28.5~29.5
	38	32.5~33.5
	44	37.0~38.0
	50	41.5~42.5

### Analysis

The arrhythmogenic properties of intramural fibrosis and endo-epicardial dissociation were investigated by analyzing the stability and complexity of chaotic electrical signals for all simulations. To effectively reflect the ability to maintain sustained signals, a simulation was evaluated to be able to maintain AF when the electrical signals were stable on the tissue more than 10 s. Therefore, all simulations were classified as stable and non-stable disorders on the basis of their time duration. Complexity was assessed by tracking phase singularities (PSs) of the membrane potential distribution, inspecting electrogram morphologies, and detecting intramural re-entry circuits and breakthrough events.

A PS is a mark of the tip of a spiral wave. The phase *θ* at each site $\vec{r}$ and time *t* is defined by the following equation:

$$\theta (t)=\arctan2\left[V_{myo}\left(t+\tau \right)-V_{mean},V_{myo}\left(t\right)-V_{mean}\right]$$ (5)

where *V*_*myo*_(*t*) is the transmembrane potential at site $\vec{r}$, *V*_*mean*_ is the average of *V*_*myo*_ during the whole time, and *τ* is a time window of 30 ms. Phase values lie between −*π* and *π*. The location of a PS was detected by the algorithm proposed by Iyer and Gray [26]. Therefore, the tip trajectory of a spiral wave was obtained by tracking the dynamic locations of phase singularities at each 1 ms. Considering that the movement speed of a PS was less than 1 m/s, if the distance between two PSs at time *t* and *t*+1 ms was less than 1 mm, these two PSs were labelled as the same identification number.

Pseudo-unipolar electrograms at different points of the endocardial and pericardial surfaces were simulated according to uniform intracellular anisotropic resistivity [27]. Therefore, the extracellular potential Φ_*e*_ was computed based on the approximation for a large unbounded volume conductor given by the following equation [28]:

$$\Phi_{e}(r)=-K\int_{vol}\nabla V_{myo}(r^{\prime })\cdot \nabla \frac{1}{R}dv$$ (6)

where ∇*V*_*myo*_ is the spatial gradient of the transmembrane potential *V*_*myo*_; *K* is a constant associated with the ratio of the extracellular and intracellular conductivity tensors; *R* is the distance from the source point $\vec{r}$ to the measuring point $\vec{r^{\prime }}$; and *dv* is the differential volume. A bipolar electrogram was then calculated by taking the difference between the two local unipolar electrograms with an interelectrode distance of 6 mm. The simulated ECG was reconstructed by subtracting two unipolar electrograms recovered from points 3 cm above the centers of the endocardium and epicardium sheets. All signals were recorded at 1 kHz and evaluated by Fourier power spectrum analysis.

**Fig 4:**
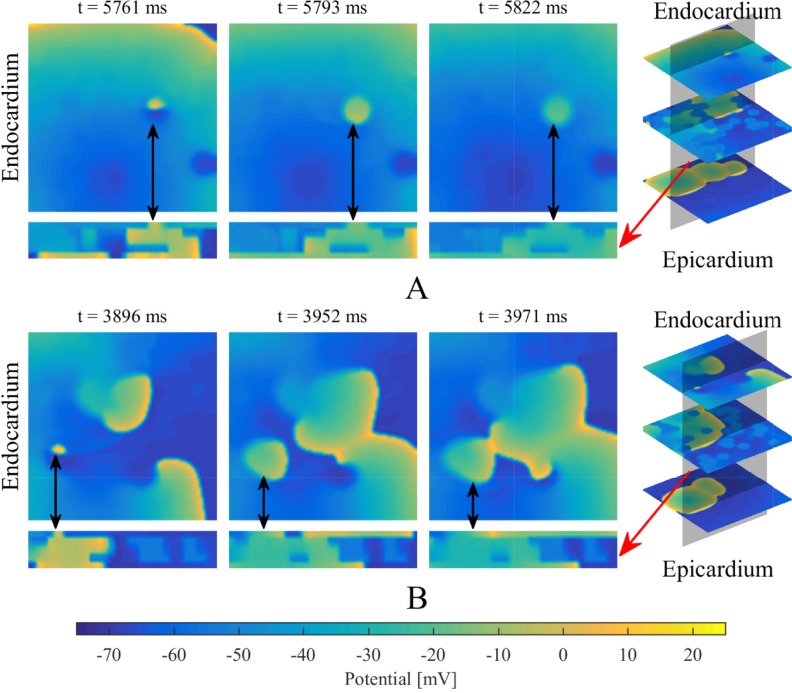
The definition of a breakthrough. (A) A wave (marked with black arrows) is annihilating in a very short time. (B) A breakthrough (marked with black arrows) produces a long enough propagation to affect other waves in the same layer. Red arrows denote the longitudinal section of the multi-layer model.

Breakthrough waves were identified to appear in the endocardium or epicardium sheet by penetrating through any myocardial patch in the isolation layers and being unconnected with the propagation of other waves in the same layer. A veritable breakthrough was required to produce a long enough propagation to affect the propagation of other waves in that layer, rather than being annihilated in a very short time, as shown in Fig 4. An intramural re-entry was a circulating action potential wave propagating inside the intramural fibrotic region. Firstly, a part of the intramural re-entry anchored to the endocardium and epicardium was directly observed as regular breakthroughs at fixed locations. Secondly, the intramural re-entry was determined if a wave was measured to repetitively activate along an anchored circuit inside the intramural fibrotic region.

Statistical tests were used to analyze the data and test reasonable hypotheses. For the stochastic group, eight datasets were extracted, including the first 28 and last 28 simulations after ranking based on dissociation degree, the first 28 and last 28 simulations after ranking based on intramural fibrosis degree, the first 20 and last 20 non-stable simulations after ranking based on dissociation degree, and the first 20 and last 20 non-stable simulations after ranking based on intramural fibrosis degree. The Kolmogorov-Smirnov test and independent sample t-test were performed to test the datasets from the stochastic group, the fibrosis-degree-controlled groups, and the dissociation-degree-controlled groups. *P*<0.05 was considered as statistically significant.

## Results

### Non-stable disorder, sustaining spiral waves, and intramural re-entry

**Fig 5:**
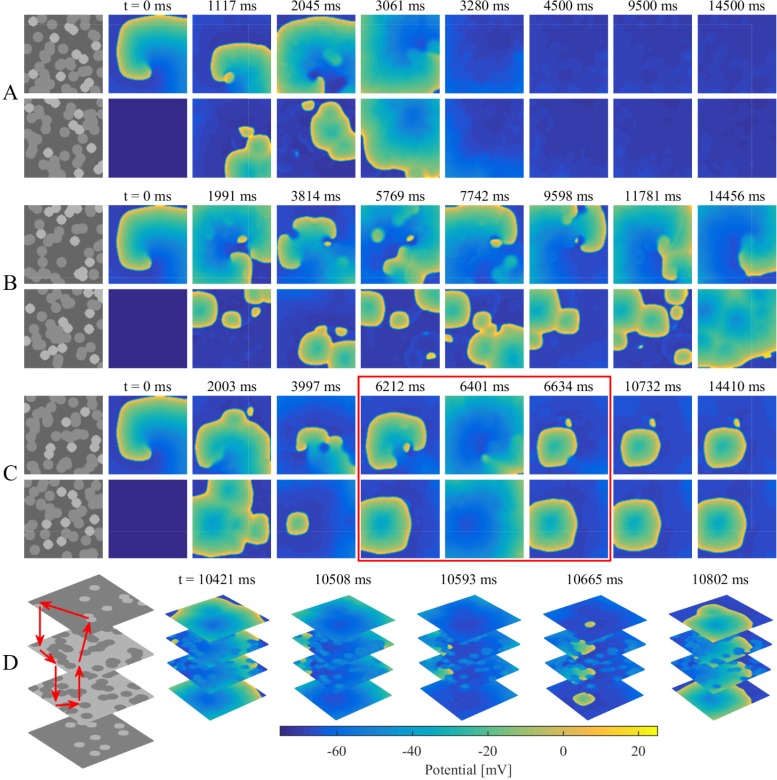
Examples demonstrating three different categories of transmembrane potential maps in the stochastic group. (A) A non-stable disorder with the rotor center of spiral wave moving away from the endocardium sheet after colliding with breakthroughs. (B) A stable disorder with spiral waves sustaining on the endocardium sheet even with collisions of breakthroughs. (C) A stable disorder maintaining with an intramural re-entry after spiral wave disappearing during collisions of breakthroughs as shown in the red box. (D) Circuit diagram of the intramural re-entry in (C). Red arrows denote the loop lines of intramural re-entry.

For all simulations of the stochastic group, three different categories of transmembrane potential maps were presented in Fig 5A–5C and the corresponding movies were provided as supplementary material (S1 Movie, S2 Movie, and S3 Movie). AF terminated when the rotor center of spiral wave moved away from the endocardium sheet after colliding with breakthroughs (Fig 5A). By contrast, spiral waves could still be maintained on the endocardium sheet even with massive collisions of breakthroughs (Fig 5B). AF persisted when an intramural re-entry appeared after the disappearance of spiral waves during collisions of breakthroughs (Fig 5C). A circuit diagram of the intramural re-entry is shown in Fig 5D. Although only stable breakthroughs were observed on the endocardium and epicardium sheets, the wave of action potential propagated along a stable re-entrant circuit inside the intramural wall due to intramural fibrosis and endo-epicardial dissociation.

### Intramural fibrosis and endo-epicardial dissociation

**Fig 6:**
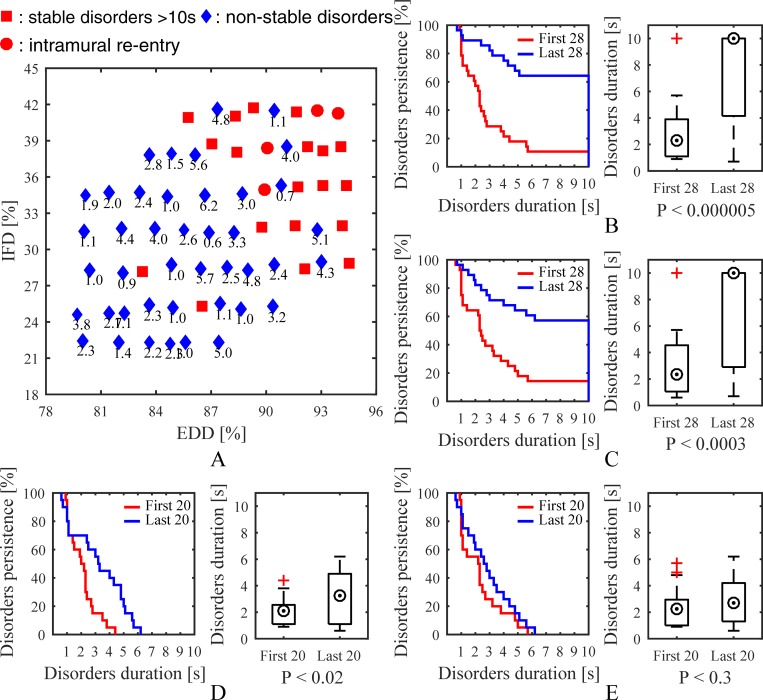
Simulation results of the stochastic group. (A) A time map of 65 simulations showing effects of intramural fibrosis and endo-epicardial dissociation on stability. Kaplan-Meier curves, boxplots, and P-values show differences between (B) the first 28 and last 28 simulations after ranking based on endo-epicardial dissociation degree, (C) the first 28 and last 28 simulations after ranking based on intramural fibrosis degree, (D) the first 20 and last 20 non-stable simulations after ranking based on endo-epicardial dissociation degree, and (E) the first 20 and last 20 non-stable simulations after ranking based on intramural fibrosis degree. *IFD* is the intramural fibrosis degree and *EDD* is the endo-epicardial dissociation degree.

Fig 6A illustrates a time map of the stochastic group. The total number of simulations in the stochastic group was 65, including 42 non-stable and 23 stable disorders. Four simulations were observed with intramural re-entry, and 21 of 23 stable disorders were distributed in the upper right corner of the time map indicating high endo-epicardial dissociation or high intramural fibrosis. Kaplan-Meier curves, boxplots, and P-values are presented in Fig 6B–6E. *P*<0.000005 suggested that there was an obvious difference between the results of simulations with high and low endo-epicardial dissociation. Similarly, *P*<0.0003 suggested an obvious difference between the results of simulations with high and low intramural fibrosis. Significantly simulations with high endo-epicardial dissociation and high intramural fibrosis were more likely to obtain stable episodes of AF.

**Fig 7:**
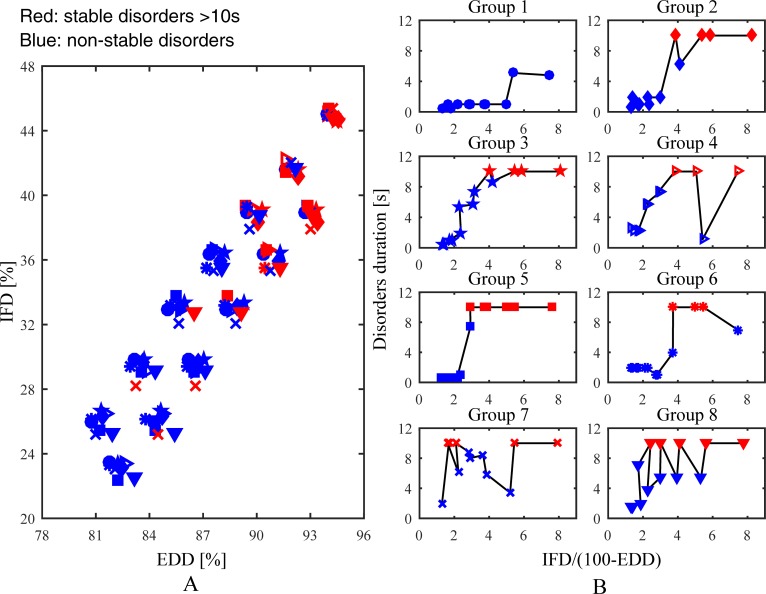
Simulation results of the interrelated groups. (A) A time map of 104 simulations shows effects of intramural fibrosis and endo-epicardial dissociation on stability. (B) Time curves of eight groups show effects of the increase of intramural fibrosis and endo-epicardial dissociation on stability. *IFD* is the intramural fibrosis degree and *EDD* is the endo-epicardial dissociation degree.

Eight interrelated groups were generated for analysing the effect of the gradual change of intramural microstructure on AF maintenance. A time map of 104 simulations in the interrelated groups is shown in Fig 7A. Overall, simulation results of the interrelated groups were consistent with the stochastic group. Time curves of the interrelated groups are shown in Fig 7B. These groups were divided into two different categories: (1) the duration of chaotic electrical signals maintaining on the tissue mainly increased with increasing intramural fibrosis and endo-epicardial dissociation, such as Group 1, 2, 3, 5, and 6; (2) the slight increase of intramural fibrosis and endo-epicardial dissociation accelerated the disappearance of chaotic electrical signals, such as Group 4, 7, and 8. Hence, some slight changes of intramural microstructure may have an unanticipated impact on the propagation of the action potential wave, reflecting the complex role of intramural microstructure in AF stability.

**Fig 8:**
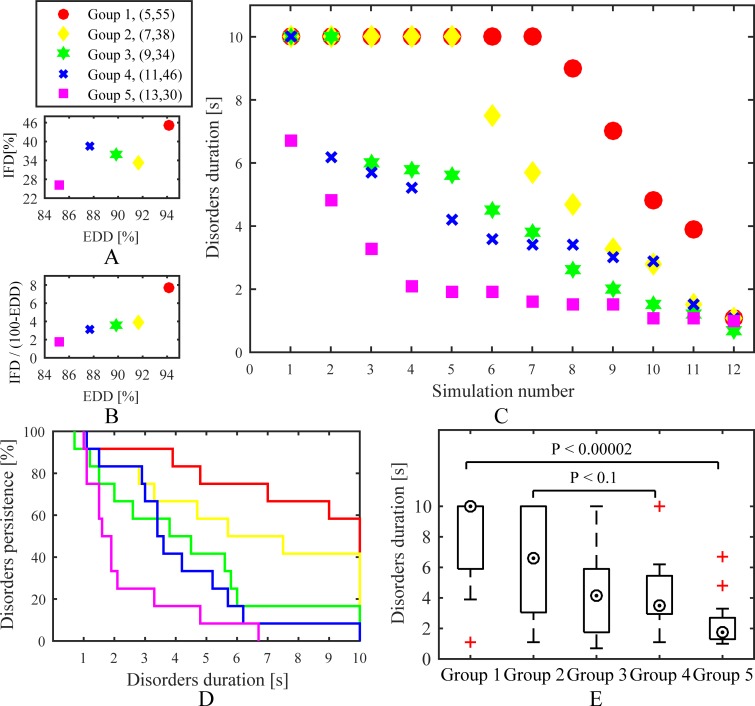
Simulation results of the fibrosis-degree-controlled groups. (A) and (B) Distributions of average of intramural fibrosis degree and endo-epicardial dissociation degree for all simulations in each group. (C) A time distribution for all simulations. (D) Kaplan-Meier curves and (E) boxplots show effects of intramural fibrosis and endo-epicardial dissociation on stability. *IFD* is the intramural fibrosis degree and *EDD* is the endo-epicardial dissociation degree.

**Fig 9:**
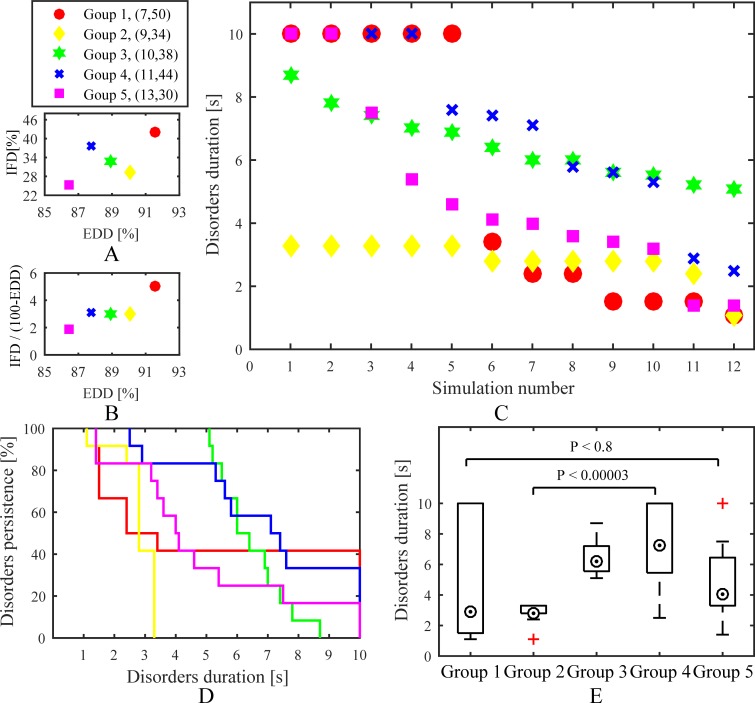
Simulation results of the dissociation-degree-controlled groups. (A) and (B) Distributions of average of intramural fibrosis degree and endo-epicardial dissociation degree for all simulations in each group. (C) A time distribution for all simulations. (D) Kaplan-Meier curves and (E) boxplots show effects of intramural fibrosis and endo-epicardial dissociation on stability. *IFD* is the intramural fibrosis degree and *EDD* is the endo-epicardial dissociation degree.

Five fibrosis-degree-controlled groups and five dissociation-degree-controlled groups were used to have a contrast effect. Comparing to the fibrosis-degree-controlled groups, all simulations of each dissociation-degree-controlled group were designed to have the same myocardial blobs in isolation layers. A time distribution, Kaplan-Meier curves, and boxplots for the fibrosis-degree-controlled groups are presented in Fig 8C–8E. The simulations with high endo-epicardial dissociation and intramural fibrosis could often obtain a longer duration of chaotic electrical signals maintaining on the tissue. Moreover, the comparison between Goup 2 and Group 4 suggested that simulations with high endo-epicardial dissociation may have more opportunities for a concomitant increase in AF stability than high intramural fibrosis. Overall, simulation results of the fibrosis-degree-controlled groups were consistent with the stochastic group. A time distribution, Kaplan-Meier curves, and boxplots for the dissociation-degree-controlled groups are presented in Fig 9C–9E. Compared to the fibrosis-degree-controlled groups, simulation results of the dissociation-degree-controlled groups presented a state of disorder with the changes of intramural fibrosis and endo-epicardial dissociation. Even with low intramural fibrosis and low endo-epicardial dissociation, simulations with some distributions of myocardial patches still had more opportunities for AF stability, such as Group 5. Conversely, Group 2 with low intramural fibrosis but high endo-epicardial dissociation failed to maintain episodes of AF.

### Phase singularities, electrogram characteristics, and breakthroughs

**Fig 10:**
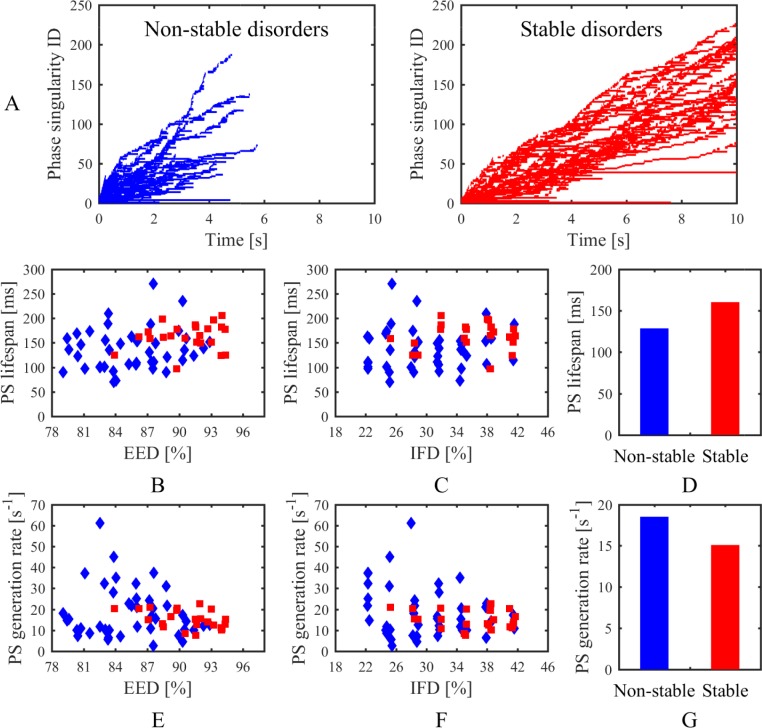
Lifespan of phase singularities in the stochastic group. (A) PS lifespans during the whole time in non-stable and stable disorders. Average lifespan of phase singularities for each simulation related to (B) endo-epicardial dissociation and (C) intramural fibrosis. (D) Average lifespan of phase singularities for all simulations in non-stable and stable disorders. Average generation rate of phase singularities for each simulation related to (E) endo-epicardial dissociation and (F) intramural fibrosis. (G) Average generation rate of phase singularities for all simulations in non-stable and stable disorders. Red squares denote stable disorders. Blue diamonds denote non-stable disorders. *IFD* is the intramural fibrosis degree and *EDD* is the endo-epicardial dissociation degree.

**Fig 11:**
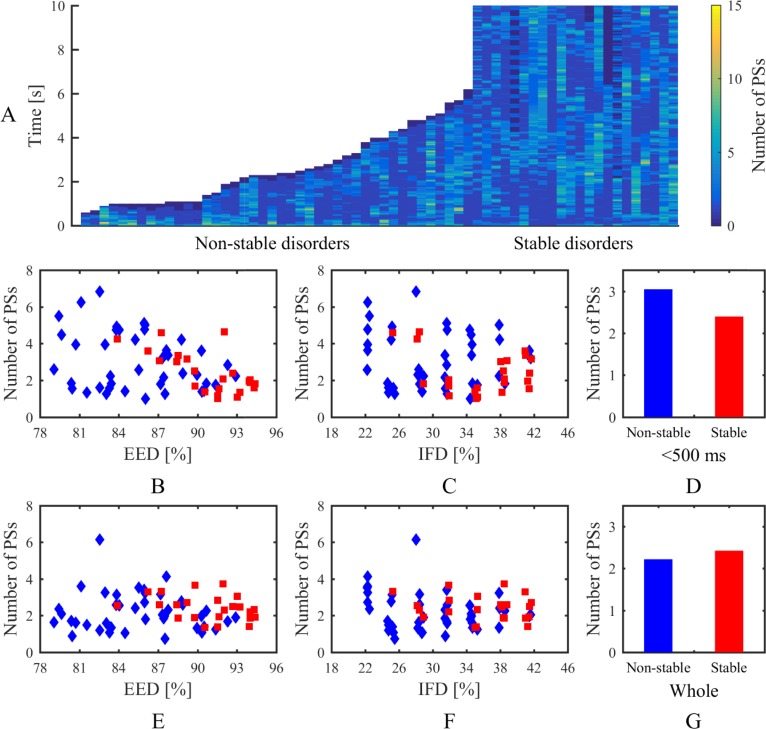
Number of phase singularities at each 1 ms in the stochastic group. (A) PS number map in non-stable and stable disorders. Average number of phase singularities during the first 500 ms for each simulation related to (B) endo-epicardial dissociation and (C) intramural fibrosis. (D) Average number of phase singularities during the first 500 ms for all simulations in non-stable and stable disorders. Average number of phase singularities during the whole time for each simulation related to (E) endo-epicardial dissociation and (F) intramural fibrosis. (G) Average number of phase singularities during the whole time for all simulations in non-stable and stable disorders. Red squares denote stable disorders. Blue diamonds denote non-stable disorders. *IFD* is the intramural fibrosis degree and *EDD* is the endo-epicardial dissociation degree.

An overview of phase singularities in the stochastic group is presented in Figs 10 and 11. Lifespan and number of phase singularities during the whole time were tracked for analysing the dynamic of spiral waves. As shown in Fig 10A, most of the PSs lasted for a short time. The average PS lifespans of stable disorders were mainly concentrated in the range of 150 ms to 180 ms and slightly increased with increasing endo-epicardial dissociation (Fig 10B and 10C). Conversely, the average PS lifespans of non-stable disorders were geographically dispersed and not linked to one another (Fig 10B and 10C). The PS generation rate was calculated by the rate between the PS amount and the whole time that atrial fibrillation persisted, reflecting the continuous replacement of new PSs. Distinctly, the PS generation rate decreased with increasing endo-epicardial dissociation (Fig 10E). Overall, the stable simulations had a longer average lifespan and smaller average generation rate of phase singularities than the non-stable simulations (Fig 10D and 10G). The activities of spiral waves varied over time on the tissue, with the changes of PS number at each 1 ms as shown in Fig 11A. During the first 500 ms, there was a downturn in average number of phase singularities at each moment with increasing endo-epicardial dissociation, especially for the stable simulations (Fig 11B). The non-stable simulations have more phase singularities at each moment during the first 500 ms (Fig 11D), whereas the stable simulations presented an overall alteration in the number of phase singularities at each moment during the whole time (Fig 11G).

**Fig 12:**
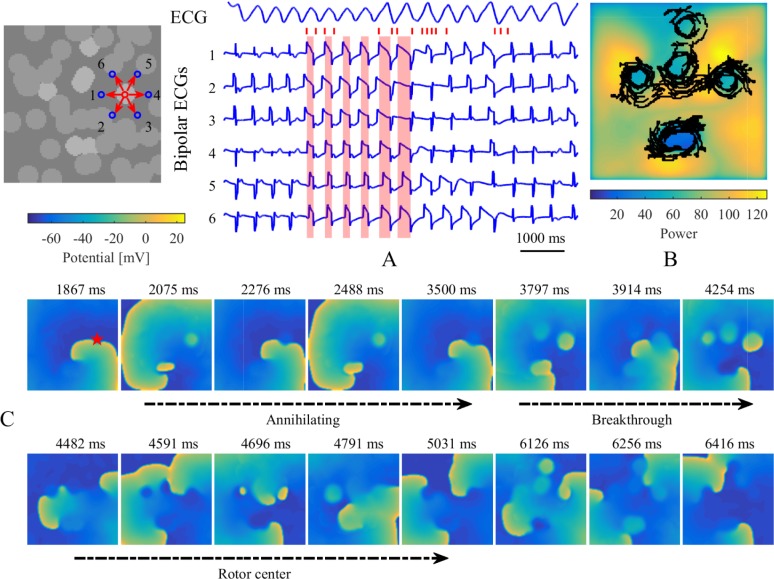
Electrogram characteristics and PS trajectories for a stable simulation without intramural re-entry. (A) Simulated ECG and bipolar electrograms recorded around a myocardial patch on the endocardium sheet. Blue circles and red arrows denote the recording sites and directions of bipolar electrograms. Bipolar electrograms covered in red shadows represent some sections from the local maximums to local minimums of No. 1 bipolar electrograms. Red tags successively denote the corresponding times of (C) transmembrane potential maps in the endocardium sheet. Red star denotes this patch location on the transmembrane potential maps. (B) The maximum map of power spectrum for every unipolar electrogram. Black lines denote the trajectories of phase singularities during the whole time.

**Fig 13:**
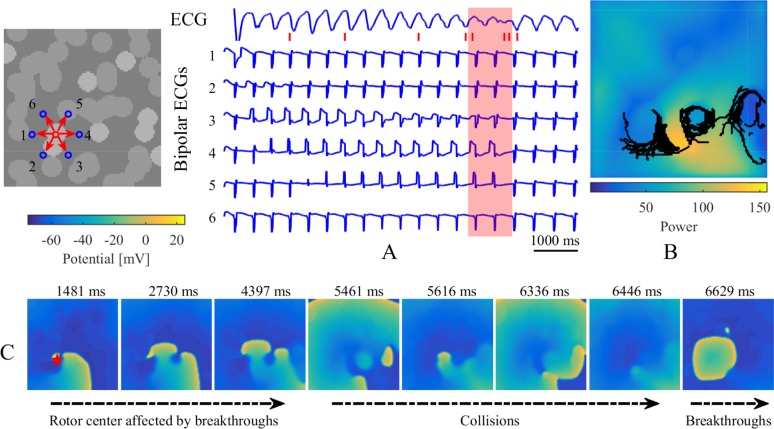
Electrogram characteristics and PS trajectories for a stable simulation with intramural re-entry. (A) Simulated ECG and bipolar electrograms recorded around a myocardial patch on the endocardium sheet. Blue circles and red arrows denote the recording sites and directions of local bipolar electrograms. Bipolar electrograms covered in red shadow represent the fractionated part of the ECG. Red tags successively denote the corresponding times of (C) transmembrane potential maps in the endocardium sheet. Red star denotes this patch location on the transmembrane potential maps. (B) The maximum map of power spectrum for every unipolar electrogram. Black lines denote the trajectories of phase singularities during the whole time.

Figs 12 and 13 show typical examples of electrogram characteristics and PS trajectories for stable simulations without and with intramural re-entry. Accordingly, bipolar electrograms around a myocardial patch presented complex variations during three different processes, including annihilating waves, breakthroughs, and rotor centers. The widths of sawtooth waves grew large as shown by the red shadows in Fig 12A. The PS trajectories encircled myocardial patches, accompanied by larger maximums of power spectrums for the unipolar electrograms recorded around myocardial patches (Figs 12B and 13B). In the case without intramural re-entry, the simulated ECG manifested as a disorderly fibrillation wave, which suggested that chaotic electrical signals varied over time on the tissue. Specifically, the ECG was fractionated during the disappearance of spiral waves with collisions of breakthroughs. After the formation of intramural re-entry, stable breakthroughs were observed on the endocardium and epicardium sheets, and the ECG transformed into a rapid and orderly wave.

**Fig 14:**
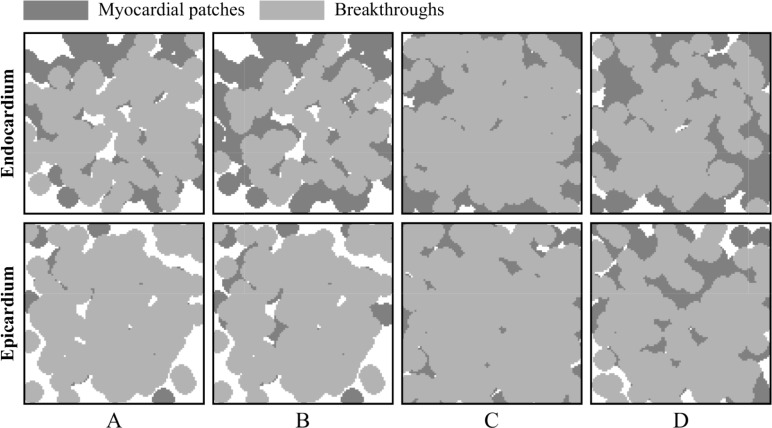
Location distributions of breakthroughs in the stochastic group. The results are extracted from (A) 23 stable simulations during the whole duration, (B) 23 stable simulations during the first 3 seconds, (C) 42 non-stable simulations, and (D) 27 non-stable simulations with the duration less than 3 seconds.

Fig 14 shows the location distributions of breakthroughs in the stochastic group. At a holistic level, breakthroughs are the behavior results of atrial intramural microstructure affecting transmural conduction on the endocardial and epicardial surfaces. The comparison between results of the stable and non-stable groups suggests that there are no significant location characteristics for breakthroughs to maintain episodes of AF.

## Discussion

In the present study, we evaluated the role of intramural fibrosis and endo-epicardial dissociation in AF stability by utilizing our multi-layer computational model. Stable intramural re-entries were observed in four simulations. A complex intramural microstructure with high intramural fibrosis and endo-epicardial dissociation could easily maintain AF episodes. In the meantime, there were differences between the effects of intramural fibrosis and endo-epicardial dissociation on the wave-dynamics of AF.

### Evidence supporting intramural re-entry

Clinical and experimental studies have suggested that AF drivers represent re-entry, ectopic foci, rotor, random wavelets, CFAEs, endo-epicardial breakthroughs, and a complex mixture of all these mechanisms [4]. Stable intramural re-entrant circuits were first demonstrated by a simultaneous epi-endocardial optical mapping study in coronary perfused ex vivo human right atrial specimens [13]. A limited number of stable intramural microanatomic re-entries in localized regions are hypothesized as the potential drivers of AF [15]. Due to the 2D limitations of clinical surface electrode mapping and 3D complexity of atrial microstructure, intramural re-entries are only partially observed as the form of periodic surface re-entries or breakthroughs on the endocardial and epicardial surfaces. Thus, this new hypothesis requires a large number of clinical experiments and computational modeling studies for resolving many controversies. Existing simplified computational models [16, 17] have not addressed intramural re-entries because of a lack of transmural atrial microstructure. The latest 3D computational high-resolution framework [18] combining the intact and entire 3D human atrial structure may be used to quantitatively analyze structural substrates, but further studies resolving tremendous computational resources are still needed.

**Fig 15:**
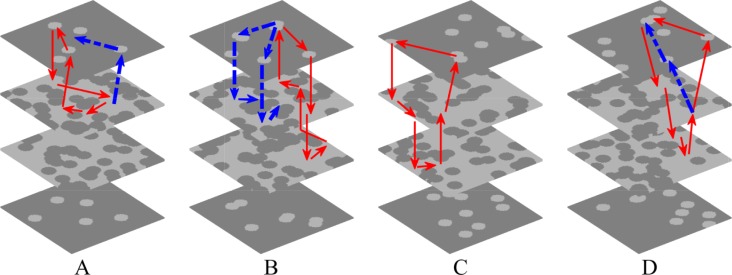
The intramural re-entry circuits of four stable simulations. The number of myocardial blobs in each layer and the number of fibrotic blobs in each layer are (A) 5, 50, (B) 7, 50, (C) 9, 42, and (D) 9, 46. Red arrows denote the loop lines of intramural re-entries, and blue arrows denote other possible conduction pathways.

By combining the simplified simulation of transmural atrial microstructure composed of endo-epicardial dissociation and intramural fibrosis, our proposed multi-layer 3D computational model was used to quantitatively analyze the effect of the complex atrial microstructure on the stability of AF. Stable intramural re-entries were observed after spiral waves disappearances during collisions of breakthroughs in four of 65 simulations in the stochastic group. The disappearance moments of spiral waves in these four stable simulations were respectively 4.3, 9.3, 6.4, and 2.2 s. As shown in Fig 15, fibrillatory conduction could steadily emanate from a complete re-entrant circuit throughout the atrial transmural wall. All of these four intramural re-entrant circuits were complete but exoteric, since there were other possible pathways to affect the stability of the conduction process. Furthermore, these simulations with intramural re-entries possessed characteristics of high endo-epicardial dissociation and intramural fibrosis, as shown in Fig 6A, which suggests that the occurrence and maintenance of intramural re-entries requires a complex atrial microstructure. Although there might be more intramural re-entrant circuits with continuous conduction of other stable simulations, more cases are required to further analyze the substrate of intramural re-entries.

### Role of intramural fibrosis and endo-epicardial dissociation in sustaining AF

The importance of a complex atrial microstructure for determining arrhythmia dynamics was recognized by experimental studies using high-resolution simultaneous endo-epicardial mapping approaches [13, 14, 29, 30]. In an atrium with structural remodeling, endomysium fibrosis in epicardium reduces the continuity of the epicardial layer and the connectivity of the trabecular meshwork, resulting in electrical activity difference and frequent transmural conduction between the endocardium and epicardium [14]. Intramural fibrosis and endo-epicardial dissociation are not generally considered to be two structurally independent concepts because of their complicated relationship. Based on our analyses, we propose that there might be a small but clear difference in function between intramural fibrosis and endo-epicardial dissociation. Intramural fibrosis primarily contributes to the formation of a complex transmission network in the atrial wall, while endo-epicardial dissociation is intuitively converted to breakthroughs on the endocardial and epicardial surfaces. In our multi-layer 3D computational model, intramural fibrosis was simulated by some fibrotic patches randomly scattered in the myocardial tissue of the fibrotic layers, while endo-epicardial dissociation was simulated by some myocardial patches randomly scattered in the fibrotic tissue of the isolation layers. Therefore, we could flexibly regulate distributions of fibrosis and myocardial patches to qualitatively analyze the role of the intramural fibrosis and endo-epicardial dissociation underlying the stability and complexity of AF.

In this study, four different simulation trial and control groups were applied for contrast research, including a stochastic group, interrelated groups, fibrosis-degree-controlled groups, and dissociation-degree-controlled groups. Lifespan and number of phase singularities for all simulations in the stochastic group were computed to visualize electrical activity during AF. Generally, simulation results of all groups demonstrated that a complex intramural microstructure with high endo-epicardial dissociation and high intramural fibrosis could be significantly beneficial to maintaining the stable episodes of AF. On a micro level, subtle changes of endo-epicardial dissociation and intramural fibrosis could affect the propagation of the action potential wave (Fig 7B). The comparison between fibrosis-degree-controlled groups and dissociation-degree-controlled groups further suggested some distributions of connection areas could be more likely to maintain episodes of AF despite the lower intramural fibrosis and endo-epicardial dissociation (Figs 8 and 9). With the increase of endo-epicardial dissociation, the average lifespans of phase singularities during the whole time slightly increased (Fig 10B), the PS generation rate decreased (Fig 10E), and the average number of phase singularities at each moment during the first 500 ms decreased (Fig 11B), especially for the stable simulations. In consequence, the endo-epicardial dissociation had a more profound effect on the electrical activity during AF than intramural fibrosis. Also, throughout the location distributions of all breakthroughs in the stochastic group, no breakthroughs with specific locations were found in the stable and non-stable groups (Fig 14).

### Study limitations

Although incorporating a simplified simulation of intramural microstructures, our multi-layer model could not establish a ground truth representation [31] for atrial tissue with thickness variation, fiber orientation, and transmural fibrosis. To focus on the qualitative analysis of intramural fibrosis and endo-epicardial dissociation, the endocardium and epicardium layers were designed with no distributions of fibrotic patches. The computational precision of our study is limited, as for the sake of computational efficiency, it is based on hexahedral elements of size 0.4 × 0.4 × 0.4 mm^3^ and a 3D slab of 40 × 40 × 3.6 mm^3^. The simulated ECG is restricted to represent the electrical activity of a piece of atrial tissue in limited time, and thus its waveform is different from the measured ECG with morphological variability and fragmentation during AF. The two isolation layers had the same number of myocardial blobs, and the two fibrotic layers had the same number of fibrotic blobs. These radiuses of cylindrical blobs in isolation and fibrotic layers were set to different constants, and the ionic current properties were spatially uniform in the different types of atrial tissue. These settings need to be further developed in our multi-layer model to approach the complexity of intramural microstructures in human atria. Although different radiuses of cylindrical blobs in fibrotic layers could categorize fibrosis into distinct patterns, our current study was concerned only with patchy fibrosis. Future studies will have to address the difference between the arrhythmogenic potentials of patchy fibrosis and interstitial fibrosis. Additionally, a simulation with the continuous emergence of triggers [8] due to tissue fibrosis could not be evaluated to maintain AF; this is because the ionic current properties in our model were too artificial to demonstrate the effect of triggers due to tissue fibrosis on sustaining AF.

## Conclusions

Here we describe a novel multi-layer computational model. To our knowledge, this is a more feasible model authentically simulating endo-epicardial dissociation and intramural fibrosis by utilizing different distributions of fibrosis and myocardial patches in a multi-layer 3D slab composed of the epicardium, connection wall, and endocardium. Simulations demonstrated that a complex intramural microstructure with high intramural fibrosis and endo-epicardial dissociation significantly increased the probability of the maintenance of AF episodes, and endo-epicardial dissociation was one of the key factors on the dynamics of electrical activity during AF. The occurrence of intramural re-entry further suggested the importance of adding a complex intramural microstructure to the AF substrate.

## Supporting information

S1 Movie One non-stable disorder in a 3D slab of atrial tissue. One non-stable disorder with the rotor center of spiral wave moving away the endocardium sheet after colliding with breakthroughs.
(AVI)

S2 Movie Sustaining spiral waves in a 3D slab of atrial tissue. One stable disorder with spiral waves sustaining on the endocardium sheet even with collisions of breakthroughs.
(AVI)

S3 Movie An intramural re-entry in a 3D slab of atrial tissue. One stable disorder maintaining with an intramural re-entry after spiral wave disappearing in the collisions of breakthroughs.
(AVI)
